# Regulatory T cell dysfunction and therapeutic advances in rheumatoid arthritis, psoriasis, vitiligo, and systemic lupus erythematosus

**DOI:** 10.3389/fimmu.2026.1932889

**Published:** 2026-10-26

**Authors:** Yanyang Liang, Haiyan Feng, Ying Wang, Junqin Li, Xuping Niu

**Affiliations:** Shanxi Key Laboratory of Stem Cells for Immunological Dermatosis, State Key Breeding Laboratory of Stem Cells for Immunological Dermatosis, Institute of Dermatology, The Ninth Clinical Medical School of Shanxi Medical University, Peking University First Hospital Taiyuan Hospital, Taiyuan, China

**Keywords:** CAR-Treg, low-dose IL-2 therapy, psoriasis, RA, regulatory T cells, SLE, Th17/Treg imbalance, vitiligo

## Abstract

Regulatory T cells (Tregs) are a critical immunosuppressive subset of CD4+ T cells, whose lineage stability and suppressive function rely on the transcription factor *FOXP3*. Within inflamed tissue microenvironments, Tregs maintain immune homeostasis and preserve tissue integrity via multiple mechanisms, including secretion of anti-inflammatory cytokines such as IL-10 and TGF-β, CD39/CD73-mediated adenosine metabolic pathways, and CTLA-4-dependent cell-contact inhibition. Nevertheless, in autoimmune diseases including systemic lupus erythematosus, psoriasis, rheumatoid arthritis, and vitiligo, Tregs frequently exhibit reduced frequency, impaired suppressive capacity, and conversion to pro-inflammatory phenotypes, accompanied by excessive activation of Th17 cells. This results in severe disruption of the Th17/Treg immune axis, which drives chronic inflammation and tissue damage. In terms of therapeutic strategies, low-dose IL-2 selectively expands functional Tregs to restore systemic immune balance. Rapamycin reverses Treg depletion and dysfunction by inhibiting the mTOR pathway, thereby reconstructing an immune-tolerant tissue microenvironment. Furthermore, engineered CAR-Treg cells exert antigen-specific anti-inflammatory effects, offering a novel therapeutic option for autoimmune diseases. Although these interventions have demonstrated promising efficacy in preclinical and early clinical studies, their clinical translation is hindered by major bottlenecks, such as precise targeting of inflamed tissues, sustained stability of Treg function under inflammatory conditions, and avoidance of systemic immunosuppressive risks. This paper systematically reviews the regulatory mechanisms and targeted therapeutic advances of Tregs in autoimmune diseases linked to Th17/Treg imbalance, analyzes the limitations of current research, and provides theoretical evidence for precision immunotherapy of autoimmune diseases.

## Introduction

### Definition of regulatory T cells

Tregs are commonly defined as CD4**^+^**CD25**^+^**FOXP3**^+^**CD127**^−^** T cells, and they are a subpopulation of T lymphocytes committed to suppress immune responses and preserve immune homeostasis (1). The absence of FOXP3-expressing Tregs leads to the development of severe autoimmunity (1–3).

### Phenotypes of regulatory T cells

According to their developmental origin, natural Tregs are classified into thymus-derived Tregs (tTregs) and peripherally derived Tregs (pTregs) (1). tTregs mature in the thymus via T-cell receptor (TCR) recognition of self-antigens, whereas pTregs differentiate from naive CD4**^+^** T cells upon recognition of nonself antigens (2, 4). In addition, Tregs can be induced *in vitro* from naive CD4**^+^** T cells with IL-2 and transforming growth factor-β (TGF-β) (3–6). These *in vitro*-induced Tregs (iTregs) share phenotypic features with natural Tregs, but exhibit distinct differences in Treg-specific demethylation patterns and lineage stability (3). Recent studies have identified GPA33 as a robust biomarker for identifying human thymus-derived regulatory T cells (7) (tTregs). The complete demethylation of the Treg-specific demethylated region (TSDR) endows tTregs with a stably suppressive phenotype, which sustains their long-term immunoregulatory capacity. By comparison, peripheral induced Tregs (pTregs) only undergo partial demethylation at the TSDR locus. This epigenetic feature renders pTreg populations relatively unstable, and they are prone to differentiating into effector T cells under specific microenvironmental cues. Additionally, *in vitro* induced Tregs (iTregs) present fully methylated TSDR, further demonstrating that these cells possess only temporary immunosuppressive properties and poor phenotypic stability (8, 9) (**Figure 1**).

**Figure 1:**
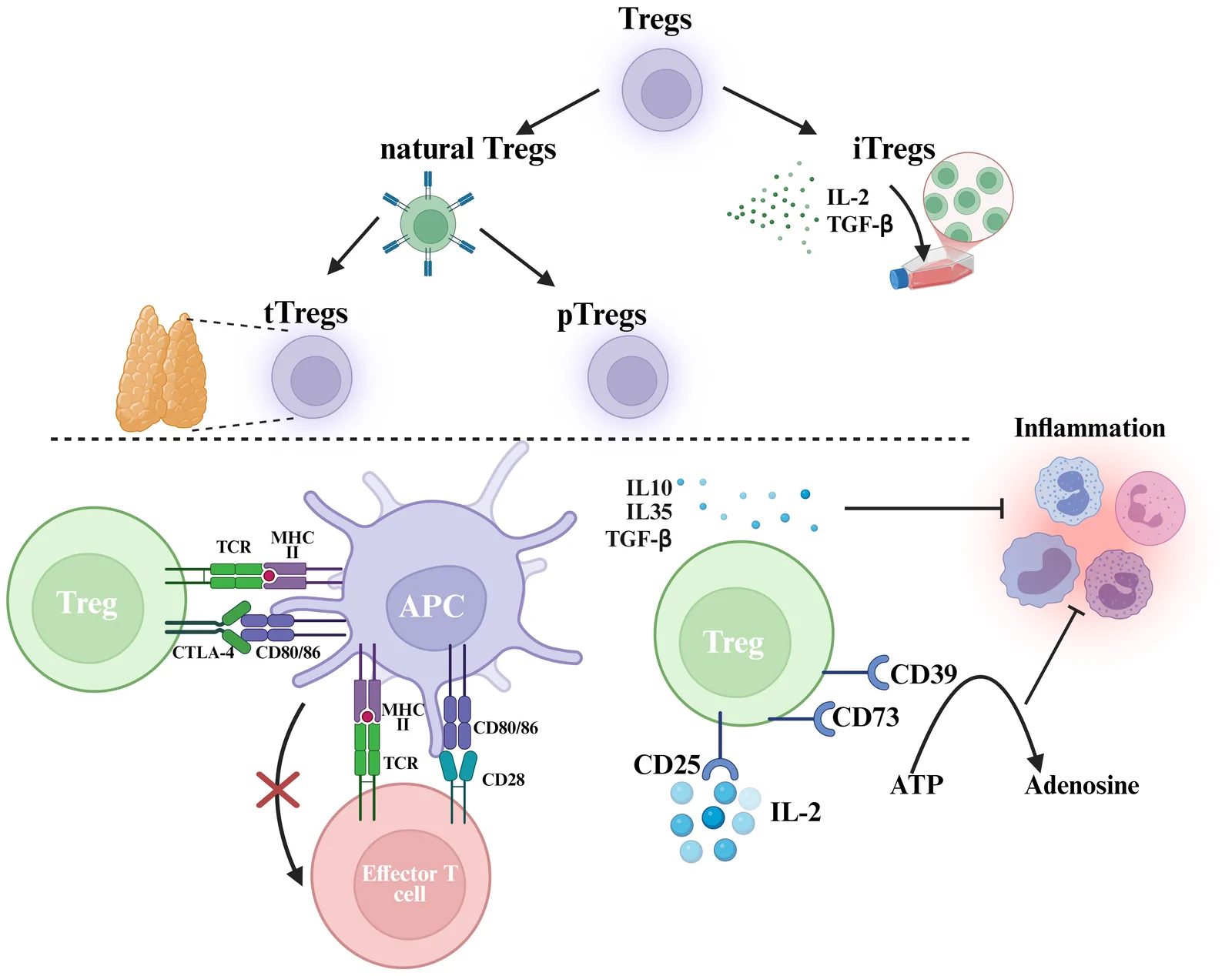
Based on their developmental origin, natural regulatory T cells (Tregs) are categorized into thymus-derived Tregs (tTregs) and peripherally induced Tregs (pTregs). In addition, naive CD4^+^ T cells can be induced to differentiate into regulatory T cells *in vitro* in the presence of interleukin-2 (IL-2) and transforming growth factor-β (TGF-β). The immunosuppressive mechanisms of Tregs fall into two main categories. In the first category, Tregs act directly on antigen-presenting cells (APCs) to impair their capacity to activate effector T cells. Specifically, the T cell receptor (TCR) on Tregs binds to antigen-MHC complexes on the APC surface, forming tight immune synapses that physically block effector T cell access to APCs. Meanwhile, cytotoxic T lymphocyte-associated antigen 4 (CTLA-4) on Tregs competitively engages CD80/CD86 on APCs, depriving effector T cells of co-stimulatory signals and directly abrogating their activation. In the second category, Tregs secrete cytokines including IL-10, TGF-β, and IL-35 to directly suppress the inflammatory activity of surrounding immune cells. Additionally, Tregs highly express CD25, which scavenges local IL-2 with high affinity and depletes this critical growth factor required for effector T cell proliferation. Tregs also convert extracellular ATP into the immunosuppressive molecule adenosine via CD39 and CD73.

In addition to the subset classification according to cellular origin, Treg cells can be further classified by their functional activation status. Human resting Tregs (rTregs) typically bear the CD45RA**^+^**FOXP3^low^ phenotype. Following activation, they differentiate into activated Tregs, which are distinguished by high expression of CD45RO, FOXP3, ICOS and CTLA-4, along with enhanced immunosuppressive potency (10). Like their human counterparts, mouse naive Tregs present a CD44^low^CD62L**^+^** phenotype and predominantly inhabit lymphoid tissues. Mouse activated Tregs (aTregs) are characterized by a CD44^high^CD62L^-^ profile and high levels of ICOS and CTLA-4. these cells migrate to inflammatory foci and mediate strong immunosuppressive effects (11).

### Markers of regulatory T cells

Molecules specifically expressed by Treg cells fall into two categories based on their locations within cells: intracellular components including FOXP3 and Helios, and extracellular surface molecules comprising CD304, CD25, CD39, CD73, CD62L, CD103, CD134, FR4, CD127^-^, CD152, CD279, GITR, CD223 and TIGIT. *FOXP3* is a vital transcription factor that drives the differentiation of naive CD4^+^ T cells and sustains Treg suppressive function (8, 12). Helios, an Ikaros family zinc finger protein, is mainly restricted to tTregs (13). The transmembrane protein CD304 (NRP-1) is also highly expressed on tTregs (14). Hence, Helios and NRP-1 are commonly utilized to separate tTregs from pTregs under physiological conditions.

CD25 is the α subunit of the IL-2 receptor and is critical for immune cell activation and proliferation. It is a standard marker for Treg identification and holds therapeutic value in both immunosuppressive and immune-activating scenarios (15). CD39 and CD73 are ectonucleases abundantly expressed by Tregs and serve as specific markers of this subset (16). The two enzymes jointly break down ATP to produce extracellular adenosine. This metabolic pathway is essential for adenosine synthesis in Tregs and further promotes the formation of an immunosuppressive microenvironment (17).

CD127 expression is low or undetectable in Tregs, which are characterized by the phenotype FOXP3^+^CD25^+^CD127 ^low^. CD134 (OX40) is a co-stimulatory receptor. It is transiently expressed on effector T cells after TCR activation, and promotes their proliferation and memory formation (18, 19).

CD62L is a selectin that promotes immune cell homing to lymphoid tissues (20). As an adhesion molecule critical to mucosal immunity, CD103 (αEβ7 integrin) regulates lymphocyte adhesion, graft rejection and the production of gut-homing effector cells (21). The two molecules jointly retain Tregs at inflammatory sites. CD152 (CTLA-4), CD279 (PD-1) and GITR are immune checkpoints highly expressed by Tregs. Their binding to ligands on effector T cells induces immunosuppression. These molecules maintain immune tolerance by inhibiting overactive immunity, yet they also promote tumor immune evasion via enhanced Treg-mediated suppression (22–24).

### Immunosuppressive functions of Tregs

Phase 1: APCs mainly reside in secondary lymphoid organs like lymph nodes (**Figure 1**). Tregs inhibit effector T cell activation by physically blocking antigen access and depleting co-stimulatory proteins on APCs. Live-cell microscopy reveals that TCRs on Tregs bind antigen-MHC complexes on APCs to form compact immunological synapses, which physically block access of effector T cells (25). When Tregs detach from APCs, antigen-MHC complexes are also removed from the APC surface, further suppressing effector T cell activity (25).

Besides interfering with antigen presentation, Tregs highly express CTLA4 to weaken the co-stimulatory signals (CD80/CD86) provided by APCs. CTLA4 competes with effector T cell CD28 to bind CD80 and CD86, and eventually removes these co-stimulatory molecules from APCs (26, 27).

Phase 2: Microenvironment Regulation

Regulatory T cells secrete a variety of anti-inflammatory cytokines and metabolic products, among which transforming growth factor-β (TGF-β) and interleukin-10 (IL-10) are the most pivotal effectors. TGF-β is broadly synthesized by diverse T cell subsets yet predominantly exists in a latent state anchored to the plasma membrane. The αvβ8 integrin expressed by Tregs can dissociate TGF-β from its inhibitory complex and convert it into the biologically active form (28). The activated TGF-β subsequently acts via an autocrine pathway to stabilize and upregulate FOXP3 expression (29).

Treg-derived IL-10 inhibits the secretion of innate pro-inflammatory cytokines including TNF, IL-6 and IL-1β, and also impairs the functional activity of antigen-presenting cells (APCs) (30). In addition, Tregs generate interleukin-35 (IL-35), a heterodimer consisting of Ebi3 and p35 subunits. This molecule alleviates inflammatory responses and enhances the immunomodulatory capacity of Tregs (31). Another core anti-inflammatory mechanism relies on the ectonucleotidases CD39 and CD73. These two enzymes function synergistically to catabolize extracellular ATP into adenosine, a molecule with potent immunosuppressive properties, thereby reinforcing Treg-mediated immune suppression (32, 33).

Apart from releasing anti-inflammatory mediators, Tregs act as a functional “IL-2 sink”. On the Treg surface, the IL-2 receptor α subunit (CD25) assembles with IL-2Rβ and IL-2Rγ to form a trimeric receptor complex (34). This complex exhibits a 100-fold greater binding affinity for IL-2 compared with the dimeric IL-2Rβ/IL-2R receptor expressed by effector T cells (35). Accordingly, Tregs scavenge IL-2 in the local microenvironment and limit the availability of this indispensable cytokine for conventional T cells (36) (**Figure 1**).

### Tregs migrate to inflamed tissues

The migration of regulatory T cells (Tregs) to inflamed tissues represents a highly coordinated active process, which can be divided into three consecutive stages. The first stage is chemotactic attraction: chemokines (e.g., CCL22, CCL20) secreted by peripheral tissues or lymphoid organs act as “chemical signals” and bind to chemokine receptors (e.g., CCR4, CCR10) on Treg surfaces to provide navigational cues for directional Treg migration (37). The second stage is vascular adhesion and transmigration. In this process, Tregs first undergo rolling, stable adhesion and transmigration across the vascular endothelium, which relies on the specific binding of integrin-family molecules (e.g., LFA-1, VLA-4) to their ligands on endothelial cells. The third stage represents tissue retention. After entering target tissues, Tregs bind to the extracellular matrix via cell-surface glycoproteins (e.g., CD44), or modulate the balance between tissue retention and egress through signaling receptors (e.g., S1PR1), ensuring sustained functional performance within target tissues (38).

Chemokine receptors (predominantly G-protein-coupled receptors) sense chemical signal gradients within the inflamed microenvironment to deliver navigational cues for directional Treg migration and trigger downstream cytoskeletal rearrangement. As a skin-homing molecule, CLA binds E-selectin and related adhesion molecules on cutaneous capillary endothelial cells, mediating the transendothelial migration and skin homing of Tregs to inflamed or lesional sites. In addition, CCR4 serves as a critical receptor for Treg migration toward inflamed sites in the skin, intestinal mucosa and lung tissues. Its core function is to respond to CCL22 (macrophage-derived) and CCL17 (dendritic cell-derived, DC) secreted by macrophages and dendritic cells (DCs) in the inflammatory microenvironment. Regarding the detailed migration mechanism: upon tissue inflammation (e.g., atopic dermatitis, allergic asthma), local immune cells secrete CCL22/CCL17 (39). Binding of these ligands to CCR4 on Tregs activates the PI3K-Akt signaling pathway, induces cytoskeletal rearrangement along the chemokine concentration gradient, and drives Treg trafficking from peripheral circulation into inflamed lesions (37). CCR10 mediates the chemotactic migration of Tregs from the dermis toward the epidermis. Responding to the CCL27 concentration gradient established by keratinocytes, it guides Tregs to localize at the dermo-epidermal junction, completing the final step of skin homing (40). Furthermore, CCR7 is an essential molecule for Treg trafficking from the peripheral circulation into secondary lymphoid organs including lymph nodes and the spleen, with its ligands being CCL19 (predominantly secreted by lymph node stromal cells) and CCL21 (highly expressed on high-endothelial venules (HEVs) within lymph nodes). Upon entering lymph nodes, CCR7**^+^** Tregs suppress naive T-cell activation via contact-dependent mechanisms (38), such as CTLA-4 binding to CD80/CD86 on DCs, thereby preventing systemic autoimmune responses. In patients with CCR7 deficiency, Tregs fail to migrate into lymph nodes, resulting in abnormally elevated Treg numbers in peripheral circulation accompanied by a sharp reduction of intra-lymph node Tregs, which ultimately leads to severe autoimmune manifestations resembling systemic lupus erythematosus (41). CXCR4 mediates Treg migration toward the bone marrow, tumor microenvironment, and certain inflamed tissues such as joint synovium. CXCL12, secreted by bone marrow stromal cells or tumor/inflammatory stromal cells, recruits Tregs to inflammatory sites (42).

### Stability and plasticity of regulatory T cells

The stability of regulatory T cells is defined as their ability to maintain FOXP3 expression under inflammatory conditions and resist transformation into pro-inflammatory effector cells. In short, stable Tregs can sustain the FOXP3**^+^** phenotype and avoid differentiating into FOXP3^-^ ex-Treg cells (43–45). Research has indicated that FOXP3 ubiquitination and degradation regulated by the E3 ligase STIP1 homology and U-box-containing protein 1 (STUB1) promote the conversion of Tregs to the Th1 phenotype, whereas Stub1 inhibition enhances Treg immunosuppressive activity.

FOXP3 expression and Treg stability are governed by multiple molecular mechanisms, including transcription, translation, epigenetic modifications, and posttranslational regulation. For example, transcription factors such as signal transducer and activator of transcription 5 (STAT5), nuclear factor of activated T cells (NFAT), and forkhead box O1 (FOXO1) directly bind to the FOXP3 promoter and enhancer and modulate its expression. Hypermethylation of the CpG motifs in the CNS regions of the FOXP3 gene locus negatively affects FOXP3 expression and stability. Histone acetyltransferases (HATs) and histone deacetylases (HDACs) further regulate FOXP3 chromatin accessibility by adjusting the acetylation levels of histone lysine residues. Furthermore, a range of E3 ubiquitin ligases (e.g., STUB1 and TNF receptor-associated factor 6 [TRAF6]), co-chaperones (e.g., 70-kDa heat shock proteins [HSP70s]), and deubiquitinases (e.g., ubiquitin-specific protease 7 [USP7], USP22, and USP47) regulate Treg stability and function by modulating the ubiquitination of FOXP3. Beyond intrinsic cellular factors, the effect of the microenvironment on Treg stability has attracted increasing attention. Microenvironmental cytokines (e.g., IL-2, IL-6, interferon-γ (IFN-γ)), metabolites (glucose, lactate, various amino acids), and antigenic components all play critical roles in regulating Treg stability. Recent studies have focused on how metabolic reprogramming modulates Treg stability. For instance, moderate activation of the mammalian target of rapamycin (mTOR) pathway promotes Treg function, whereas excessive mTOR signaling and elevated glycolysis reduce FOXP3 expression and impair Treg suppressive capacity (45).

Treg cells do not constitute a functionally homogeneous population. In response to cytokine signals from the local microenvironment, they can remodel their transcriptional programs and functional profiles, and differentiate into distinct Th-like subsets. This plasticity enables Tregs to exert more targeted suppression against specific types of immune responses (46, 47). Multiple Th-like Treg subsets have been discovered to date. Among them, Th1-like Tregs are distinguished by the co-expression of T-BET, CXCR3 and FOXP3 (48, 49). Other identified populations include Th2-like Tregs (GATA3^+^IRF4^+^IL-4^+^FOXP3^+^) (50), RORγt^+^ Tregs that concurrently produce IL-17A and FOXP3 (51), as well as follicular Tregs (Tfr-like Tregs) with the phenotype of BCL6^+^FOXP3^+^ (52) (8). Additionally, under the influence of distinct cytokines, Tregs can be reprogrammed into various Th-like regulatory T cell subsets. Specifically, IL-12, IL-27 and IFN-γ induce the formation of Th1-like Tregs; IL-2, IL-4, IL-13 and IL-33 promote the differentiation of Th2-like Tregs; Th17-like Treg generation is driven by IL-1β, IL-6, IL-17, IL-21 and IL-23; and Tfr-like Treg differentiation is co-induced by IL-6 and IL-21 (8).

Th1-like Tregs secrete IFN-γ and exhibit elevated expression of Th1 signature molecules including T-BET and CXCR3 (48), and they specifically suppress Th1-type immune responses (53). Th2-like Tregs exert potent inhibitory effects on Th1 and Th17 immune responses (50, 54). They also possess enhanced migratory ability, which allows them to rapidly accumulate in tumor tissues and foster a tumor-permissive microenvironment to promote immune escape (50). Th17-like Tregs express IL-17, IL-22, IL-23R and RORγt (51, 55). This subset is predominantly detected in the intestinal tract of both humans and mice (51). Their mechanisms of action remain unclear, and they may exert pathogenic effects in inflammatory bowel disease and colon cancer. As for Tfr-like Tregs, these cells are defined by co-expression of BCL6 and FOXP3. Their primary function is to modulate immune reactions inside germinal centers (GCs), thereby sustaining immune tolerance and B cell homeostasis (56, 57). Taken together, the biological functions of Th-like Tregs have not been fully elucidated, and their ultimate functional properties depend on the local tissue microenvironment, transcription factor profiles, and the stability of FOXP3 expression. Notably, Treg plasticity exerts dual effects. On the one hand, it enables precise targeted suppression of specific Th cell immune responses by differentiating into distinct Th-like Treg subsets. On the other hand, such plasticity may also cause Tregs to lose their immunosuppressive function and even acquire a pro-inflammatory phenotype, contributing to disease progression in certain pathological contexts. Therefore, when evaluating the immunoregulatory effects of Tregs, their specific suppressive effects on Th subsets must be balanced against the potential pro-inflammatory risk mediated by plasticity.

### Th17/Treg imbalance

Th17 cells and regulatory T cells (Tregs) are two key subsets derived from CD4^+^ T cell differentiation, playing opposing proinflammatory and anti-inflammatory roles in immune homeostasis: Th17 cells drive inflammatory responses by secreting cytokines such as IL-17, whereas Tregs maintain immune tolerance by producing suppressive factors including IL-10 and TGF-β. In autoimmune diseases, Th17/Treg imbalance is one of the core mechanisms driving disease onset, progression and even relapse. The differentiation of Th17 cells and Tregs is intimately linked. TGF-β alone induces Treg differentiation, whereas TGF-β combined with IL-6 (or IL-21, IL-23) skews differentiation toward the Th17 lineage. Therefore, the relative levels of proinflammatory cytokines (e.g., IL-6) and TGF-β collectively determine the Th17/Treg balance; in autoimmune diseases, this balance is disrupted, with Th17 cells gaining predominance and Tregs failing to adequately control inflammation (58). Retinoid-related orphan receptor γt (RORγt) binds to the promoter regions of Th17-associated cytokine genes, enhancing the transcription of IL-17, IL-22 and other cytokines. In parallel, it activates downstream signaling pathways that promote the differentiation of naive CD4^+^ T cells into Th17 cells while suppressing the expression of FOXP3, the key Treg transcription factor, thereby disrupting the Th17/Treg balance and skewing immune responses toward inflammation (59).

Upon activation, the phosphatidylinositol 3-kinase-protein kinase B (PI3K-Akt) signaling pathway enhances the expression and activity of RORγt, a key Th17 transcription factor, by phosphorylating a series of downstream targets, thereby promoting Th17 cell differentiation and development (60). Meanwhile, it suppresses the stability and activity of FOXP3 in Tregs, attenuating their immunosuppressive function. Furthermore, this pathway can also affect Treg metabolism and survival, further impairing their regulation of immune responses and ultimately leading to Th17/Treg imbalance (61).

In addition to cytokines and transcription factors, metabolic factors also participate in the regulation of Th17/Treg imbalance. Unsaturated fatty acids (e.g., ω-3 fatty acids) can suppress Th17 differentiation and promote Treg generation, whereas saturated fatty acids may promote Th17 polarization, exerting adverse effects on the Th17/Treg balance (62). Furthermore, Th17 differentiation and function depend on glycolysis for energy and metabolic intermediates, whereas Tregs rely mainly on fatty acid oxidation to maintain their function (63). therefore, alterations in glucose metabolism can affect the balance between the two subsets. Age, sex, hormone levels and environmental factors (e.g., diet, drugs and stress) can also influence the Th17/Treg balance (64, 65).

In autoimmune diseases, abnormal chemokine gradients within the skin microenvironment hinder the recruitment of circulating Tregs to inflamed lesions and impair the retention of *in situ* skin-resident Tregs (66). Aberrant activation of metabolic pathways further reduces the stability of FOXP3 expression in Tregs and weakens their immunosuppressive function. Accordingly, impaired stability and defective skin-homing capacity of tissue-resident Tregs in lesions disrupt local Th17/Treg homeostasis through multiple mechanisms, including dysregulated chemokine signaling and cellular metabolic reprogramming.

## The role and mechanism of Tregs in disease

### Rheumatoid arthritis

Rheumatoid arthritis (RA) is a chronic inflammatory autoimmune disorder, which is predominantly manifested as persistent synovial inflammation in joints and ultimately leads to joint damage and dysfunction (57). Although the pathogenesis of RA is intricate and involves the interplay of multiple factors, the roles of regulatory T cells (Tregs) in disease initiation and progression have attracted increasing attention. Treg cells from RA patients are characterized by numerical reduction and functional impairment (67, 68). Beyond the immunosuppressive capacity of Tregs, their overall efficacy also relies on their ability to migrate toward inflamed tissues.

### Dysfunction of Tregs

In the hypoxic synovial microenvironment of RA, HIF-1α is highly expressed and stabilized. HIF-1α is one of the few proteins known to directly bind FOXP3 and promote its degradation. In the hypoxic synovial microenvironment, HIF-1α interacts with FOXP3, stimulates FOXP3 ubiquitination, and consequently induces FOXP3 degradation via the proteasome pathway, ultimately impairing Treg suppressive function (67, 69). These cells fail to suppress the production of pro-inflammatory cytokines such as TNF-α and IFN-γ by CD4^+^ T cells and monocytes, and their capacity to inhibit the proliferation of effector T cells is also diminished (67). These findings indicate that Treg dysfunction, rather than Treg deficiency, plays a more critical role in the pathophysiology of RA. In the collagen-induced arthritis (CIA) mouse model, CD25 depletion exacerbates the severity of collagen-induced RA, while adoptive transfer of CD4^+^CD25^+^ T cells can effectively alleviate disease symptoms (70) (**Table 1**).

**Table 1:** The role of Tregs in diseases and the underlying mechanisms.

Disease	Peripheral Treg count	Th17/Treg Imbalance	Treg homing to inflamed tissues	Treg plasticity	Other mechanisms
Rheumatoid arthritis (RA)	decrease	Elevated IL-6 shifts the Th17/Treg balance toward a pro-inflammatory phenotype.	Increased IL-6 reduces VASP phosphorylation, ultimately impairing the homing capacity of Tregs.	N/A	HIF-1α binds FOXP3, promoting its ubiquitination and proteasomal degradation and thereby impairing Treg suppression.
Psoriasis	decrease	The Notch1 signaling pathway regulates Th17/Treg imbalance in plaque psoriasis.	Significantly decreased expression of CCR4, CCR5, and CXCR3 impairs the directional migration of Tregs to cutaneous inflammatory lesions.	Upon *in vitro* stimulation, Tregs can differentiate into IL-17A-secreting Th17-like Tregs.	Upregulated IL-6 and miR-210 inhibit Treg immunosuppressive functions. Tregs from plaque psoriasis patients exhibit reduced CD73 expression and attenuated immunosuppressive potency.
Vitiligo	decrease	The Th17/Treg balance shifts toward a pro-inflammatory phenotype.	Impaired CCR6 expression hinders Tregs migration and aggravates depigmentation in mouse models.	Regulatory T cells can differentiate into IFN-γ-secreting Th1-like Tregs.	Impaired secretion of IL-10, TGF-β, and IL-35, together with reduced FoxP3 and CTLA-4, diminishes Treg immunosuppressive capacity.
Systemic lupus erythematosus (SLE)	decrease	IL-6 diverts TGF-β signaling toward STAT3-RORγt axis, promoting excessive Th17 differentiation and disturbing Th17/Treg balance.	Reduced expression of IL-2 receptor (CD25) on Treg cells impedes IL-2 signaling and impairs Treg proliferation and migration.	N/A	Decreased CTLA-4 expression and reduced levels of the anti-inflammatory cytokines IL-2 and IL-10 weaken the suppressive function of Tregs.

VASP, Vasodilator-stimulated phosphoprotein.

### Th17/Treg imbalance

Numerous studies have demonstrated that the Th17/Treg ratio is shifted toward the Th17 cell lineage in patients with rheumatoid arthritis (RA) relative to healthy controls (68, 71, 72). Characterized by ROR-γt expression and the production of IL-17 family cytokines, Th17 cells exert particularly vital effects on RA pathogenesis (73). By secreting IL-17A, IL-17F and IL-22, Th17 cells stimulate synovial fibroblasts and macrophages to robustly produce IL-1, IL-6, TNF-α and PGE2, which further aggravate synovial inflammation. These cells also prompt synovial stromal cells and innate lymphoid cells to secrete GM-CSF, thereby initiating and exacerbating joint inflammation (74–76). Elevated IL-6 levels are detected in the peripheral blood and synovial fluid of RA patients. This cytokine accelerates inflammatory responses by inducing neutrophils to generate reactive oxygen intermediates and proteolytic enzymes, as well as promoting osteoclast differentiation through RANKL-independent pathways (77). Notably, IL-6 not only suppresses Treg differentiation but also facilitates Th17 cell development. This dual effect shifts the Treg/Th17 balance toward a pro-inflammatory phenotype (58) (**Table 1**).

### VASP regulates Treg migration

Vasodilator-stimulated phosphoprotein (VASP) is a cytoskeletal effector protein. It coordinates the recruitment of monomeric actin to the barbed ends of actin filaments, blocks actin filament capping, and participates in linear actin polymerization and filament bundle formation. For these reasons, VASP serves as a critical regulator of cell migration (58). IL-6 can reduce the phosphorylation level of VASP, an essential modulator of T cell migration. This alteration hinders the recruitment of Tregs to inflamed sites and ultimately impairs their immunosuppressive functions (78) (**Table 1**). Elevated IL-6 levels in the synovial fluid of RA patients reduce VASP phosphorylation, impairing Treg recruitment to inflamed joints and resulting in inadequate local immunosuppression. This allows sustained activation of CD4^+^ effector T cells and macrophages, driving TNF-α-, IFN-γ- and RANKL-mediated synovial inflammation and bone erosion (78). Notably, tocilizumab, an IL-6 receptor antagonist already used in RA treatment, significantly increases the proportions of resting Tregs (rTregs) and activated Tregs (aTregs) (79).

### Psoriasis

Psoriasis (Ps) is a chronic immune-mediated skin disorder characterized by hyperproliferation of keratinocytes, infiltration of inflammatory cells and accumulation of T lymphocytes. Accumulating evidence has demonstrated that regulatory T cells (Tregs) exert critical effects on the pathogenesis of psoriasis (80, 81).

### Dysfunction of regulatory T cells

Recent studies have verified that Tregs isolated from most psoriasis patients display obvious functional defects. CD4^+^CD25^high^FOXP3^+^ Tregs harvested from lesional skin or peripheral blood fail to suppress the activation and proliferation of effector T cells (82, 83). Tregs derived from peripheral blood of pediatric psoriasis patients also exhibit impaired suppressive capacity. Multiple mechanisms contribute to the compromised inhibitory function of lesional Tregs in psoriasis. Specifically, exposure to high concentrations of IL-6 diminishes Treg activity (84). In addition, overexpressed miR-210 inhibits FOXP3 expression, reduces the secretion of IL-10 and TGF-β, and elevates the production of IFN-γ and IL-17A (85). Impaired adenosine signaling pathways may also lead to the loss of immunosuppressive function in Tregs from psoriasis patients. Research conducted by Yan et al. revealed that Tregs from patients with plaque psoriasis show markedly decreased CD73 expression accompanied by inactivated CD73/AMPK signaling, which further weakens their immunosuppressive potency (86) (**Table 1**).

### Impaired homing capacity of Tregs to inflamed lesions

Apart from defective suppressive function, inefficient homing of Tregs to inflammatory sites also impairs the overall function of Tregs in psoriasis patients. In patients with psoriasis, the expression of skin-homing receptors (e.g., CLA, CCR4, CCR5) on Tregs is significantly downregulated, impairing their directed migration to inflamed skin. In contrast, CCR7 expression is markedly upregulated on psoriatic Tregs, with enhanced chemotactic responses to its ligands CCL19/CCL21, indicating that Treg migration is redirected from inflamed skin to secondary lymphoid organs. Impaired Treg homing to skin lesions precludes the establishment of an effective immunosuppressive microenvironment, leaving effector T cells (e.g., Th17) unrestrained and thereby exacerbating lesional inflammation and promoting disease progression (66, 87).

### Imbalance between Th17 cells and regulatory T cells

A series of studies have reported an aberrant Th17/Treg ratio in psoriasis patients (74, 77, 88, 104). Circulating Th1 and Th17 cell frequencies are increased, whereas the numbers of Th2 cells and Tregs are reduced. Research from Ma’s group indicated that the Th17/Treg imbalance in plaque psoriasis is regulated by the Notch1 signaling pathway (89), and this pathological imbalance could be reversed via dual antiplatelet therapy (**Table 1**). Currently, biologics targeting the IL-23 and IL-17 pathways have become the mainstream approach for correcting Th17/Treg imbalance by suppressing Th17 cell survival and effector function. In contrast, strategies that directly expand Tregs, such as low-dose IL-2, provide a new direction for restoring immune tolerance by enhancing immunosuppressive capacity and promoting the survival and expansion of FOXP3^+^ Tregs.

### Regulatory T-cell plasticity

In psoriasis, skin-resident Tregs exposed to IL-6, IL-1β and TGF-β acquire Th17-like features via STAT3 activation and RORγt upregulation, converting into Th17-like Tregs (90). These cells co-express FOXP3 and RORγt, and a high RORγt/FOXP3 ratio drives the proinflammatory IL-17A transcriptional program, exacerbating local cutaneous inflammation. Although therapies targeting IL-17 or IL-23 suppress effector Th17 cell activity, they cannot fundamentally reverse the pathogenic reprogramming that Tregs have already undergone (91). Therefore, future therapeutic strategies should target Treg stability itself rather than merely blocking downstream effector cytokines (**Table 1**).

### Vitiligo

Vitiligo is a common depigmenting skin disorder characterized by aberrant immune attack against melanocytes, which leads to localized loss of skin pigment and formation of white macules (92). Given the prominent visible lesions, vitiligo exerts severe adverse impacts on patients’ psychological well-being.

### Reduced frequency and dysfunction of Tregs in vitiligo

Accumulating evidence has verified that the counts of regulatory T cells (Tregs) in both lesional skin and peripheral blood are markedly decreased in vitiligo patients compared with healthy controls (93, 94). Circulating Treg levels are further lower in patients with early-onset vitiligo (aged 1–20 years) than those with late-onset disease (95, 96). Tregs suppress aberrant activation of effector T cells via secretion of inhibitory cytokines including IL-10, TGF-β and IL-35. In the context of vitiligo, the production of these suppressive cytokines is substantially impaired, and such cytokine imbalance is closely correlated with disease stage and lesion extent. The concentration of IL-10 within vitiligo lesions is significantly reduced relative to non-lesional skin (97), and patients with active vitiligo exhibit lower IL-10 levels than those with stable disease. Consistently, diminished serum TGF-β levels are positively associated with disease duration and the percentage of affected body surface area (**Table 1**).

### Treg plasticity and Th1-like Tregs

Recent single-cell RNA sequencing studies have detected aberrant IFN-γ production by Tregs in vitiligo lesions. Chen et al. further identified that IFN-γ^+^ Tregs in vitiligo patients are Tbet^+^CXCR3^+^FOXP3^+^ Th1-like Tregs, which display enhanced chemotactic responses to CXCL10 and CXCL16 (98). Moreover, healthy donor-derived canonical Tregs can be converted into Th1-like Tregs upon exposure to vitiligo patient serum, indicating that the Th1-skewed inflammatory milieu in vitiligo drives pro-inflammatory phenotypic switching of Tregs (98). These Th1-like Tregs exhibit markedly impaired suppression of CD8^+^ T cell proliferation and activation, representing a critical link in the immune dysregulation underlying vitiligo (**Table 1**).

### Impaired skin homing capacity of Tregs

The crosstalk between chemokine ligands and receptors precisely orchestrates directional migration of Tregs. Overexpression of CCL22 in the skin of vitiligo-prone mice alleviates depigmentation and augments cutaneous infiltration of CCR4^+^ Tregs (99). Conversely, CCR6 deficiency impairs Treg migration to the skin, thereby reducing local suppression and exacerbating depigmentation (100). In addition, single-cell RNA sequencing (scRNA-seq) identifies a CCL5-CCR5 signaling-mediated chemotactic network linking CD8^+^ effector T cells and Tregs within vitiligo lesions. Surface-expressed CCR5 on Tregs guides their recruitment to CCL5-secreting CD8^+^ T cells and facilitates intercellular crosstalk (101). Targeted modulation of these chemokine axes recruits circulating Tregs and restores cutaneous immune tolerance, representing a promising therapeutic strategy for vitiligo (**Table 1**).

### Dysregulated expression of transcription factors

FOXP3 expression is markedly reduced in peripheral blood and lesional skin of vitiligo patients relative to healthy individuals, with further lower FOXP3 levels observed in active patients than stable counterparts, confirming that aberrant FOXP3 expression is a critical driver of impaired suppressive function of Tregs in vitiligo (102). Auxiliary transcription factors such as NFAT also govern Treg lineage differentiation and functional stability. The Ca²^+^–calmodulin–calcineurin–NFAT signaling axis exhibits reduced activation in Tregs from vitiligo patients, which downregulates the expression of FOXP3, IL-10 and CTLA-4, attenuates Treg immunosuppressive activity. Ultimately, they fail to effectively restrain autoreactive CD8^+^/CD4^+^ T cells, resulting in melanocyte destruction and driving vitiligo progression (102).

### Systemic lupus erythematosus

Systemic lupus erythematosus (SLE) is an autoimmune disorder characterized by broken immune tolerance and persistent autoantibody production. Major CD4^+^ T helper subsets in SLE, including Th1, Th2, Th17, regulatory T cells and follicular helper T cells, exhibit quantitative and functional abnormalities. CD4^+^ T cells from SLE patients are capable of providing B cell help, overproducing IL-17 while secreting insufficient IL-2, and infiltrating target tissues (103).

### Reduced quantity and functional defects of Tregs

The number of regulatory T cells (Tregs) in peripheral blood and tissues is markedly decreased in patients with systemic lupus erythematosus (SLE), accompanied by impaired capacity to suppress the activation and proliferation of effector T cells (Teffs) (104, 105). Decreased CTLA-4 expression has been validated in SLE patients and correlates with disease activity, indicating that disrupted CTLA-4 signaling exacerbates Treg dysfunction. Collectively, these data identify CTLA-4 as a potential therapeutic target for SLE (106). Adoptive transfer of Tregs into lupus-prone mice alleviates inflammation and attenuates tissue injury, highlighting the therapeutic potential of Tregs (107) (**Table 1**).

### Th17/Treg imbalance

Patients with SLE exhibit elevated levels of Th17-associated cytokines including IL-17, IL-23 and IL-6, which establish an inflammatory microenvironment supporting Th17 differentiation and persistence. In contrast, anti-inflammatory cytokines essential for Treg development and maintenance, such as IL-2 and IL-10, are frequently downregulated. This cytokine imbalance blunts the suppressive function of Tregs and enhances effector T cell activation, thereby amplifying systemic inflammation (108). Consistent with this observation, Gali et al. reported that serum IL-17 and IL-6 concentrations are significantly higher in SLE patients than healthy controls, and both cytokines positively correlate with disease activity (109). Transforming growth factor-β (TGF-β) acts as a master switch balancing Th17 and Treg lineage differentiation (110). In isolation, TGF-β drives the generation of FOXP3^+^ Tregs via SMAD2/3 signaling. However, in the presence of IL-6, TGF-β signaling redirects toward STAT3 activation, upregulates RORγt and initiates Th17 differentiation (111). Accordingly, abundant IL-6 in SLE skews TGF-β signaling toward Th17 polarization and aggravates immune imbalance. IL-2 also serves a critical regulatory role: it induces FOXP3 expression and inhibits Th17 differentiation to sustain Th17/Treg homeostasis (112). Dysregulated expression of FOXP3 and RORγt further disturbs this balance and sustains persistent inflammatory responses (**Table 1**).

### Aberrant signaling pathways IL-2 signaling is indispensable for Treg development and function

SLE patients display disordered IL-2 production and reduced expression of the IL-2 receptor CD25 on Tregs, which impairs IL-2 signal transduction and compromises Treg proliferation, survival and suppressive activity (113). Additional pathways including TCR and Notch signaling are also perturbed, disrupting Treg activation and migratory capacity (114). Impaired Treg function leads to aberrant activation of autoreactive T cells, which provide help to B cells for the production of autoantibodies against nuclear self-components; the resulting immune complexes deposit in tissues, triggering inflammation and tissue damage. Studies have shown that low-dose IL-2 effectively restores Treg levels in patients with SLE: when added to glucocorticoid (prednisone) therapy, low-dose IL-2 markedly increased Treg levels in 92.86% of patients, whereas only 27.27% of patients receiving prednisone alone showed increased Treg levels (115).

### Treg therapy

#### *In vivo* expansion of Tregs by low-dose IL-2

Regulatory T cells (Tregs) constitutively express high-affinity IL-2 receptor complexes consisting of CD25, CD122 and CD132, whereas effector T cells mainly express intermediate-affinity IL-2 receptors without CD25. Low-dose IL-2 selectively activates high-affinity IL-2 receptors, induces STAT5 phosphorylation, promotes Treg proliferation and enhances immunosuppressive function, with no obvious activation of effector T cells.

Given this unique mechanism, low-dose IL-2 (Ld-IL-2) therapy can selectively expand Tregs and restore immune tolerance, emerging as a promising therapeutic strategy for systemic lupus erythematosus (SLE) (116, 117). A recent multicenter, double-blind phase IIb clinical trial enrolled 152 patients with active SLE, who were randomized into four groups at a 1:1:1:1 ratio. Patients received subcutaneous injection of 0.2 MIU, 0.5 MIU, 1.0 MIU Ld-IL-2 or placebo on alternate days for 12 weeks, followed by once-weekly administration for another 12 weeks. This study demonstrates that Ld-IL-2 exerts dose-dependent therapeutic effects for SLE, and 1.0 MIU is identified as the optimal dose. The sequential regimen of alternate-day administration for the first 12 weeks and weekly administration for the subsequent 12 weeks can efficiently expand Tregs and rebuild immune tolerance. This regimen improves SRI-4 and LLDAS response rates, alleviates multi-organ damage, reduces serum autoantibody levels and facilitates glucocorticoid tapering, with favorable safety profiles and a lower risk of infection (118) (**Table 2**).

**Table 2:** Treg therapy.

Disease	Ld-IL-2	CAR-Treg	Rapamycin/mTOR inhibitors
Rheumatoid arthritis (RA)	In phase II double-blind controlled trials, low-dose IL-2 (1 MIU) plus methotrexate decreased CDAI/SDAI scores, increased Treg/Th17 ratio, and inhibited pro-inflammatory cytokines (IL-17A, IFN-γ).	Among six SBT-77-7101-treated participants, four showed ≥50% reduction in swollen/tender joint counts and four achieved ≥2-point reduction in DAS28-CRP scores.	N/A
Psoriasis	One psoriasis patient (20-year disease duration) received daily subcutaneous low-dose IL-2 (0.5 MIU) for 80 days; clinical improvement occurred on day 3, with complete resolution of scaling and marked erythema alleviation by day 7.	N/A	N/A
Vitiligo	A phase II clinical trial is underway to evaluate the efficacy of IL-2-Fc mutant in patients with vitiligo (ClinicalTrials.gov identifier: NCT06113328).	GD3-specific CAR-Tregs exhibit increased IL-10 secretion, improved control of melanocyte-directed cytotoxicity, and marked delay of depigmentation in vitiligo mouse models.	A topical clinical trial (NCT05342519) is ongoing.
Systemic lupus erythematosus (SLE)	Multicenter phase IIb trials confirmed that Ld-IL-2 (1.0 MIU) is the optimal dose; intermittent administration efficiently expands Tregs in a dose-dependent manner.	CD19-specific CAR-Tregs suppress B-cell activation and reduce autoantibody titers in murine SLE models.	In a small-scale study, rapamycin combined with prednisone reduced BILAG and SLEDAI scores and permitted lower prednisone dosage in patients.

Ld-IL-2, Low-Dose Interleukin-2; CAR-Treg, chimeric antigen receptor regulatory T cell.

CDAI, Clinical Disease Activity Index; scoring system for Crohn’s disease; SDAI, Simplified Disease Activity Index; DAS28-CRP: widely-used scoring systems for assessing disease activity in RA, rheumatoid arthritis.

BILAG and SLEDAI scores: Systemic Lupus Erythematosus Disease Activity Index.

In a phase II randomized, double-blind, placebo-controlled clinical trial for rheumatoid arthritis (RA), participants were randomly assigned at a 1:1 ratio to receive either 1 MIU low-dose IL-2 plus methotrexate or placebo plus methotrexate for a 12-week treatment period, followed by a 12-week follow-up (119). The results revealed that patients in the low-dose IL-2 plus methotrexate group (n=17) exhibited markedly greater reductions in CDAI and SDAI disease activity indices from baseline compared with those in the placebo plus methotrexate group (n=23), with statistically significant intergroup differences (P = 0.018 and P = 0.015, respectively). Moreover, combination therapy with low-dose IL-2 and methotrexate (MTX) significantly elevated the Treg/Th17 ratio and reduced levels of proinflammatory cytokines including IL-17A and IFN-γ (119) (**Table 2**).

This article reports one patient with a 20-year history of psoriasis refractory to topical treatment. The patient received monotherapy with low-dose interleukin-2 (IL-2) via daily subcutaneous injection of 0.5 MIU for 80 consecutive days (120). Clinical improvement was observed as early as day 3 with marked reduction of scales; complete resolution of cutaneous scales and remarkable alleviation of erythema were achieved on day 7. The patient’s Psoriasis Area and Severity Index (PASI) score decreased from a baseline of 4 to 3 on day 3, 2 on day 7, and 0 on day 46 (120). The patient completed the full 80-day IL-2 course to sustain therapeutic efficacy. No disease relapse or adverse events occurred during the extended treatment period, and the treatment was subsequently discontinued voluntarily. No new psoriatic lesions were detected at the latest follow-up (120) (**Table 2**). This is a preliminary study, and large-scale clinical trials are still required to verify the efficacy and safety of low-dose IL-2 therapy for psoriasis.

An IgG Fc-fused CD25-targeted IL-2 mutein preferentially binds IL-2 receptors on Tregs rather than conventional naive T cells and NK cells. Studies in peripheral blood from healthy volunteers have demonstrated that this agent elevates Treg numbers in a dose-dependent manner (121). A phase II clinical trial is currently underway to evaluate the efficacy of this IL-2-Fc mutein in patients with vitiligo (ClinicalTrials.gov identifier: NCT06113328) (**Table 2**).

Low-dose IL-2 has not obtained FDA approval in the United States, fundamentally due to the lack of definitive clinical trial data supporting its safety and efficacy, as well as dual constraints from the complex biological properties of the drug and strict regulatory requirements (122, 123). First, the Treg-selective dose window is extremely narrow: Tregs constitutively express the high-affinity IL-2 trimeric receptor (CD25/CD122/CD132), whereas resting effector T cells and NK cells express only the intermediate-affinity CD122/CD132 dimer; consequently, a modest increase in dose can shift the response from Treg expansion toward activation of effector T cells and NK cells (124). Second, under inflammatory conditions, activated effector T cells upregulate CD25, such that low-dose IL-2 may concurrently expand autoreactive effector cells (125). Third, native IL-2 has an extremely short serum half-life (approximately 15–30 minutes), necessitating frequent dosing and producing unstable peak–trough concentration fluctuations (126). Fourth, and most importantly, Treg expansion does not necessarily translate into clinical benefit: controlled trials in SLE confirmed Treg expansion but formally failed to meet their predefined primary clinical efficacy endpoints; even the highly Treg-selective engineered molecule rezpegaldesleukin, despite producing a 10- to 15-fold increase in CD25^high^Tregs, did not meet the primary endpoint in its phase II SLE trial, suggesting that merely increasing circulating Treg numbers does not necessarily restore local immune tolerance (125, 127). Furthermore, validated pharmacodynamic biomarkers and standardized dosing regimens are currently lacking, and the pleiotropic effects and long-term safety of sustained polyclonal Treg expansion remain incompletely elucidated (116, 128).

Taken together, low-dose IL-2 remains unapproved not merely because of regulatory requirements but, more fundamentally, because several scientific challenges remain unresolved: the Treg-selective dose window is narrow; its serum half-life is short; its selectivity declines in inflammatory settings; and validated biomarkers and standardized dosing regimens are lacking. Nevertheless, the favorable efficacy observed in early-phase trials in diseases such as SLE and RA continues to render low-dose IL-2 a promising therapeutic strategy worthy of further development.

### Rapamycin

Rapamycin, also known as sirolimus, is a potent mTOR inhibitor. Studies have demonstrated that rapamycin elevates the proportion of Tregs while reducing pro-inflammatory Th17 cells, thereby restoring immune tolerance. In a small-scale study involving nine patients with refractory SLE, combination therapy with rapamycin and prednisone for 6–48 months produced significant reductions in BILAG and SLEDAI scores and enabled prednisone tapering (129). In addition, repeated administration of rapamycin expanded Treg populations and induced sustained disease remission in the Vitesse mouse model of vitiligo (130). The dual function of rapamycin, which suppresses effector T cells and augments inhibitory Treg responses, has motivated its evaluation in multiple clinical trials for autoimmune disorders, including an open-label trial of topical rapamycin for vitiligo (ClinicalTrials.gov identifier: NCT05342519). Nevertheless, the clinical application of rapamycin in RA and psoriasis remains exploratory. Further large-scale, multicenter clinical trials are required to validate its optimal dosage, long-term safety, and interindividual variability in therapeutic efficacy (94) (**Table 2**).

### CAR-Treg

Promising strategies for Treg research include reprogramming T cells with chimeric antigen receptor (CAR) constructs and genetic editing of Tregs via CRISPR-Cas9 technology. CAR-Treg therapy combines regulatory T cells (Tregs) with chimeric antigen receptor (CAR) technology via genetic engineering. CAR-Treg-mediated suppression of surrounding autoreactive effector T cells occurs after antigen-specific activation within target tissues, through which local immune tolerance is restored via mechanisms including the secretion of inhibitory cytokines and CTLA-4-mediated contact inhibition. CARs recognize defined molecular antigens: tissue-restricted autoantigens (such as myelin oligodendrocyte glycoprotein [MOG] in the central nervous system, citrullinated proteins in RA synovium, and ganglioside GD3 on melanocytes) or lineage markers on the surface of autoreactive B cells (such as CD19) (131, 132). Following target-antigen recognition, CAR-Tregs expand locally and secrete immunosuppressive cytokines such as IL-10 and TGF-β, suppress the proliferation of effector T cells, restore immune tolerance, and consequently block aberrant autoimmune responses (133). In 2024, Doglio et al. developed CD19-specific CAR-Tregs (Fox19 CAR-Tregs). In murine SLE models, these cells markedly suppressed B-cell activation, lowered autoantibody titers, delayed B-cell exhaustion, and restored bone marrow and splenic homeostasis without obvious adverse reactions (134). SBT-77–7101 is an autologous CAR-Treg therapy targeting citrullinated proteins (CitPs), signature antigens present in the inflamed synovium of rheumatoid arthritis. SBT-77–7101 demonstrates favorable early safety profiles. All treated participants showed good tolerability. Among six subjects across two dose cohorts, four (67%) achieved a ≥50% reduction in swollen and tender joint counts at week 4 post-treatment versus baseline. Consistently, four participants (67%) attained a clinically meaningful improvement defined as a ≥2-point decrease in DAS28-CRP, a composite index for assessing RA disease activity (142).

Multiple sclerosis (MS) is an autoimmune disease affecting the central nervous system (CNS), in which immune-mediated myelin attack leads to neuronal death. Myelin oligodendrocyte glycoprotein (MOG) is expressed on the outer surface of myelin, the insulating sheath surrounding nerves, rendering it an ideal target for CAR-Treg therapy. Targeting MOG enables CAR-Tregs to efficiently infiltrate inflamed CNS sites; upon MOG-driven CAR activation, these cells potently suppress myelin-reactive immune cells. *In vitro* studies have demonstrated that expanded MOG-CAR Tregs maintain high expression of suppressive T-cell phenotypic markers including FOXP3 and HELIOS, and exert CAR-dependent inhibitory functions toward T cells and antigen-presenting cells. Moreover, during inflammatory responses in experimental autoimmune encephalomyelitis (EAE) mice, MOG-CAR Tregs specifically traffic to the central nervous system and effectively constrain disease progression (135). Furthermore, CAR-Treg therapy represents a promising approach to resolve immune dysregulation in vitiligo. Genetically engineered CAR-Tregs expressing chimeric antigen receptors targeting melanocyte-associated antigens such as ganglioside D3 (GD3) can restore immune tolerance by modulating cytotoxicity and mitigating inflammatory responses. In pre-clinical studies using humanized mouse models of vitiligo, GD3-specific CAR-Tregs exhibited capacities to protect melanocytes, promote IL-10 production, and suppress the activity of autoreactive T cells, thereby alleviating depigmentation (42) (**Table 2**).

The core limitations of CAR-Treg clinical translation lie in phenotypic instability under inflammatory microenvironments, insufficient skin-homing efficiency, and the risk of systemic immunosuppression (136). Inflammatory tissues are enriched in IL-23, IL-6, and IL-21, which can drive Tregs to downregulate FOXP3, upregulate RORγt, and produce IL-17A, posing a risk that genetically engineered CAR-Tregs may convert into pathogenic Th17-like cells, thereby attenuating therapeutic efficacy or even exacerbating inflammation (136). To preserve the regulatory phenotype, a constitutive promoter can be inserted at the FOXP3 locus via HDR (Homology-Directed Repair), dCas9-based epigenetic editing can stabilize FOXP3 and inhibitory genes, or IL-23/IL-6 inhibitors can be combined to block these pathways (132). Regarding homing and safety, skin Tregs rely on homing receptors such as CCR4, CCR8, and CLA, and engineering these receptors can enhance the directed migration of CAR-Tregs to skin lesions; meanwhile, selecting antigens highly expressed in lesions (e.g., IL-23R and HLA-A2) enables local antigen-specific suppression, avoiding the risks of systemic immunosuppression, infection, and malignancy caused by polyclonal Tregs. Therefore, only through coordinated optimization of phenotypic stability, skin homing, and antigen specificity can the safety risks of CAR-Treg conversion into Th17-like cells and systemic immunosuppression be mitigated.

### Treg cells with functional materials

The combined application of regulatory T cell (Treg) therapy with functional biomaterials represents a cutting-edge research hotspot for the treatment of autoimmune diseases in recent years. Functional materials include microneedles, liposomes, and targeted nanoparticles. Biomaterials can markedly improve the therapeutic efficacy of Treg-based therapy by optimizing Treg delivery efficiency and enhancing their *in-vivo* stability. The team of Zhen Gu at Zhejiang University developed porous microneedles (PMNs) with favorable mechanical properties and spacious embedding chambers to support cell viability, as well as tunable channels to facilitate cell migration, for local Treg-based treatment of psoriasis. In a mouse model of psoriasis, PMN-mediated Treg therapy enhanced the anti-inflammatory response by reducing leukocyte infiltration, suppressing the expression of inflammatory cytokines, restoring immune homeostasis, and increasing the local FOXP3^+^ Treg population (42). However, this technology is still confined to preclinical investigations and early-phase clinical exploration. No mature clinical cases are available for broad application, and further validation of its safety and efficacy is warranted.

## Conclusion

Insufficient quantity or functional defects of Treg cells constitute the core pathogenesis of autoimmune diseases, which disrupt immune homeostasis and trigger disease progression. Accordingly, increasing the number and functional activity of Treg cells represents a vital therapeutic strategy for autoimmune diseases. Current Treg-targeted therapies for autoimmune diseases are mainly divided into two categories. The first category refers to targeted expansion and modification of Treg cells, including adoptive transfer of polyclonal Tregs, CAR-Treg therapy, and *in vivo* Treg expansion regimens using low-dose IL-2 and rapamycin. The second category is the combination of Treg cells with functional materials. Functional materials compensate for the poor specificity and targeting defects of conventional cell therapies, facilitate the enrichment of therapeutic components in lesion sites, and prolong their *in vivo* circulation time.

Low-dose IL-2 has shown promising efficacy in the treatment of autoimmune skin diseases. However, restricted by inaccurate dosage control, limited pharmacokinetic properties and insufficient clinical evidence, it is currently only applied as a supplementary strategy for conventional immunosuppressive therapy. Future studies are required to optimize IL-2 formulations, develop engineered IL-2 with longer half-life and higher targeting ability, and conduct large-scale clinical trials to overcome existing therapeutic limitations.

Accumulating studies have demonstrated that antigen-specific Treg therapies (e.g., CAR-Tregs) exert superior therapeutic effects compared with expanded polyclonal Tregs even at low cell doses (137). CAR-Tregs specifically accumulate in inflammatory sites and exert localized immunomodulatory functions (138). Genetic co-expression of FOXP3 with CAR constructs efficiently blocks the transdifferentiation of Tregs into Th17 cells, providing a novel therapeutic target for skin diseases driven by Th17/Treg immune imbalance (139–141). Although genetically modified Tregs have exhibited great potential in treating autoimmune and allogeneic immune diseases, accumulating clinical trials of CAR-Treg therapies indicate that additional data are still needed to fully evaluate their clinical safety and potential risks.
